# ^15^N–^13^C Dipole Couplings in Smectic Mesophase of a Thermotropic Ionic Liquid

**DOI:** 10.1007/s00723-018-1000-7

**Published:** 2018-04-19

**Authors:** M. Cifelli, V. Domenici, V. I. Chizhik, S. V. Dvinskikh

**Affiliations:** 1 Dipartimento di Chimica e Chimica Industriale, Università di Pisa, 56124 Pisa, Italy; 2 Department of Physics, St. Petersburg State University, St. Petersburg, 199034 Russia; 3 Laboratory of Biomolecular NMR, St. Petersburg State University, St. Petersburg, 199034 Russia; 4 Department of Chemistry, KTH Royal Institute of Technology, 10044 Stockholm, Sweden

## Abstract

Unique combination of ionic conductivity and anisotropic physical properties in ionic liquid crystals leads to new dynamic properties exploited in modern technological applications. Structural and dynamics information at atomic level for molecules and ions in mesophases can be obtained by nuclear magnetic resonance (NMR) spectroscopy through the measurements of dipole–dipole spin couplings. While ^13^C–^1^H and ^15^N–^1^H dipolar NMR spectra can be routinely acquired in samples with natural isotopic abundance, recording ^15^N–^13^C dipolar NMR spectra is challenging because of the unfavourable combination of two rare isotopes. In the present study, an approach to measure ^15^N–^13^C dipole-dipole NMR spectra in static liquid crystalline samples with natural abundance is introduced. We demonstrate that well-resolved spectra can be recorded within 10 h of experimental time using a conventional NMR probe and a moderately strong magnetic field. The technique is applied to a thermotropic smectic mesophase formed by an ionic liquid with imidazolium-based organic cation.

## Introduction

Ionic liquid crystals (ILC) belong to a new class of materials that are ionic liquids capable of forming liquid crystalline phases on cooling from isotropic state [1–3]. Unique feature of a material in the liquid crystalline state is that a high degree of molecular translational and rotational mobility is combined with partial orientational and positional order. ILC have the typical properties of ionic liquids and, at the same time, a nano-scale structure of liquid crystals (LC). This leads to the unique combination of ionic conductivity displayed by ionic liquids and anisotropic physico-chemical properties, revealed by liquid crystalline materials. The presence of the molecular orientational order in fluid ionic material leads to new dynamic properties exploited in modern technological applications, such as optoelectronics [4], development of organic photo-luminescent materials [5], tribology [6], and as structure directing agents in synthesis approaches [7].

Nuclear magnetic resonance (NMR) spectroscopy is a powerful experimental tool for investigating molecular conformation, orientational order, and dynamics in LC [8–11]. ^1^H NMR spectra in LC lack chemical resolution and display complex spectral shapes difficult to analyse. ^13^C NMR applied to samples with the natural isotopic abundance allows obtaining site-specific structural and dynamic information by achieving a high spectral resolution and by selective suppression/introduction of anisotropic spin interactions [9, 10]. Deuterium (^2^H) NMR can be also employed in isotopically labelled liquid crystals to gather orientational and dynamic information [8]; however, this approach requires chemically demanding and expensive synthesis of specifically deuterated LC samples [12]. ^2^H NMR spectra in samples with natural isotopic abundance (NAD NMR [11]) can also be recorded; however, resonance assignment of NAD spectra is often ambiguous [13]. Nitrogen atoms are present in many functional groups in the molecules that form thermotropic mesophases [14]. Recording NMR spectra of the abundant nitrogen isotope ^14^N with spin number *I* = 1 is often impractical due to the large quadrupolar coupling of the order of MHz and short *T*_2_ relaxation times [15]. Few examples of ^14^N NMR studies of liquid crystals in terms of ordering and dynamic properties were possible in relation with particular mesophase orientations with respect to the magnetic field and/or molecular geometries [16–19]. Turning to the ^15^N isotope (spin-1/2), its major disadvantage is poor signal intensity due to the low natural abundance of 0.37%. However, it has been recently demonstrated that the nitrogen-15 NMR in bulk LC materials at the natural abundance level (NAN15 NMR) can also be routinely recorded in non-spinning samples with a high molecular orientational order and strong anisotropic spin interactions [20].

Structural information in rigid and soft solids can be obtained by NMR spectroscopy through the measurements of dipole–dipole spin couplings, which are very sensitive to interatomic distances. The dipolar interaction is also orientation-dependent and, thus, reports on molecular dynamics at the atomic level. Most commonly, ^15^N–^1^H and ^13^C–^1^H heteronuclear dipolar couplings between abundant protons and rare isotopes are measured by various 2D separated local field (SLF) spectroscopy methods applied to samples with natural isotopic abundance [21, 22]. Experimental detection of a ^15^N–^13^C dipolar splitting at the natural abundance level is challenging due to the combination of the two rare isotopes; the fraction of ^15^N–^13^C coupled pairs is, in fact, only 4 × 10^−5^. Nevertheless, in a straightforward but long (48 h) experiment in the 5CB nematic phase, the ^13^C-satellites were observed in NAN15 spectrum. The intensity of the carbon-13 (natural abundance of 1.1%) dipolar satellites is only 0.55% of that of the central peak. In the current work, we present an approach to access ^15^N–^13^C dipolar couplings with much higher sensitivity and greater spectral resolution. We demonstrate that, with careful experimental design, the ^15^N–^13^C dipole–dipole spectra in LC samples with natural abundance of both isotopes can be acquired within hours of experimental time using the conventional commercial NMR probes and magnets. Spectra are recorded in static (non-rotating) samples with the phase director aligned in the magnetic field. In the present study, we focus on a thermotropic smectic mesophase formed by an ionic liquid with imidazolium-based organic cation [23].

## Experimental

Sample of ionic liquid mesogen 1-dodecyl-3-methyl-imidazolium tetrafluoroborate (C12mimBF4) was obtained from Prof. Saielli (University of Padova, Italy) and was used as received. The sample has the following phase transition temperatures: Isotropic $\mathop{\longrightarrow}\limits_{}^{{ + \;46\;{\kern 1pt} {}^{ \circ }{\text{C}}}}$ Smectic A $\mathop{\longrightarrow}\limits_{}^{{ + 10{\kern 1pt} \;{}^{ \circ }{\text{C}}}}$ Crystal [23]. Upon cooling from the isotropic phase in the presence of the strong external magnetic field, director of the smectic phase is aligned perpendicular to the magnetic field vector. Experiments were performed using Bruker 500 Avance III Spectrometer at Larmor frequencies of 500.1, 125.7, and 50.7 MHz for ^1^H, ^13^C, and ^15^N, respectively. Approximately 0.4 ml of the sample was loaded in a standard 5 mm NMR tube. ^15^N spectra were recorded using solution state Bruker 5 mm BBO (broadband observe) probe-head applying a single-pulse excitation (SPE) with 15 μs 90°pulse and with proton broadband (BB) decoupling at the nutation frequency of 9 kHz. A solution state triple-frequency probe ^1^H–^13^C–^15^N was used to record ^13^C spectra. The ^1^H, ^13^C, and ^15^N pulse lengths were 7, 15, and 50 μs, respectively. For heteronuclear proton decoupling in the mesophase, spinal64 sequence [24] with the ^1^H nutation frequency of 20 kHz was applied during acquisition time of 100 ms. For selective ^15^N decoupling, continuous wave (CW) irradiation with a nutation frequency of 200 Hz was used. For scans without ^15^N decoupling, decoupler carrier frequency was shifted 10 kHz off-resonance. To enhance ^13^C signals, cross-polarization (CP) was used with the contact time of 8 ms and rf fields of *γB*_1_/2*π* ≈ 13 kHz. The ^15^N chemical shift was referenced relative to liquid NH_3_ by using ^15^N resonance at 77 ppm of 0.1 M Urea solution in DMSO as an external reference [25]. The relaxation delay values and number of scans are specified in figure captions. The numerical simulation of dipolar spectra was performed using Simpson program [26].

## Results and Discussion

### Natural Abundance Nitrogen-15 (NAN15) NMR Spectra in Mesophase

**Fig. 1:**
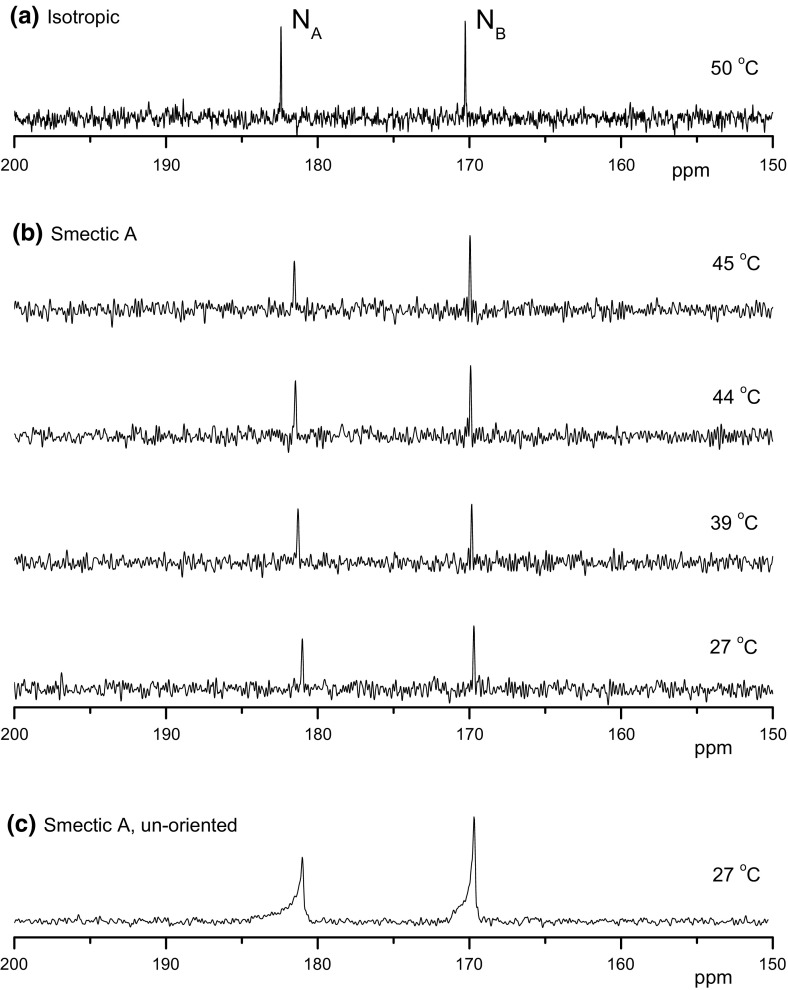
^15^N NMR spectra of C12mimBF4 in the isotropic (**a**) and smectic A phase (**b**) at indicated temperatures. For each spectrum, 64 scans were accumulated with relaxation delay of 7 s. In the case **c**, the ^15^N spectrum was recorded in the un-aligned smectic A phase at 27 °C. For this spectrum, 4096 scans were accumulated with the relaxation delay of 7 s (8 h experimental time)

Representative ^15^N NMR spectra in the smectic phase of the ionic liquid C12mimBF4 are shown in Fig. 1 along with the spectrum obtained in the isotropic phase. Spectral assignment is according to the literature data in solution [27] and is confirmed by ^13^C–^15^N dipolar NMR spectra discussed in the next section. The chemical shifts (CS) of the observed nitrogen signals in the mesophase with respect to the corresponding isotropic shifts depend on residual anisotropies of the partly averaged CS tensors. In particular, in uniaxial mesophases, the rigid-lattice CS tensor is transformed into an axially symmetric tensor with the principal elements $\delta_{||}^{\text{LC}}$ and $\delta_{ \bot }^{\text{LC}}$, corresponding to components along and perpendicular to the phase symmetry axis, respectively. The isotropic shift is then $\delta_{\text{iso}} = (\delta_{||}^{\text{LC}} + 2\delta_{ \bot }^{\text{LC}} )/3$. In the present sample, the residual chemical shift anisotropy (CSA) $\Delta \delta^{\text{LC}} = \delta_{||}^{\text{LC}} - \delta_{ \bot }^{\text{LC}}$ is in the range of a few ppm, much smaller as compared to ^15^N CSA of the imidazolium-based ions in rigid solids where the CSA values of about 200 ppm were reported [28]. This small residual CSA indicates a high degree of mobility of the flexible organic cation in the smectic layered phase. Due to the negative anisotropy of the diamagnetic susceptibility ∆*χ*, the mesophase director aligns perpendicular to the magnetic field direction and the chemical shift of the spectral lines is given by the component $\delta_{ \bot }$ of the CS tensor. This is, indeed, evidenced by the comparison to the NAN15 spectrum recorded in an un-aligned sample with random director orientation. The un-aligned sample was prepared by cooling from the isotropic phase in the absence of magnetic field (outside of NMR magnet). In this sample, the axially symmetric CSA pattern is observed and the chemical shift of the $\delta_{ \bot }$ edge coincides with the line position in the aligned sample. From the spectrum in Fig. 1c, the residual CSA is estimated to 2.7 and 1.6 ppm for nitrogens A and B, respectively.

### ^13^C–^15^N Dipolar Couplings

We have previously demonstrated that ^13^C–^15^N dipolar splittings in LC can be directly observed in ^15^N 1D NMR spectra [20]. Due to large coupling in the range of kHz for the nitrile group –C≡N in 5CB, the observed ^13^C satellites were well separated from the strong central line. The measurement, however, required a long experimental time of about 2 days to accumulate sufficient amount of scans. That long measurement would be impractical in most application studies. Besides, smaller couplings to remote carbons were not resolved in ^15^N spectra due to overlap with a broad foot of the central line. An approach, described below, provides a greater sensitivity and a higher resolution.

The technique is based on a difference spectroscopy concept previously applied in ^1^H NMR of solutions [29]. In the approach used to measure heteronuclear *J*-couplings to rare isotopes in proton solution NMR, one proton spectrum was recorded with decoupling of the rare spins and a second one was recorded without decoupling. These two spectra were subtracted and the sequence was repeated. In the ^1^H difference spectrum, the strong central resonance peak was suppressed, while the satellites due to *J*-coupling to the low abundance nuclei were observed [29].

**Fig. 2:**
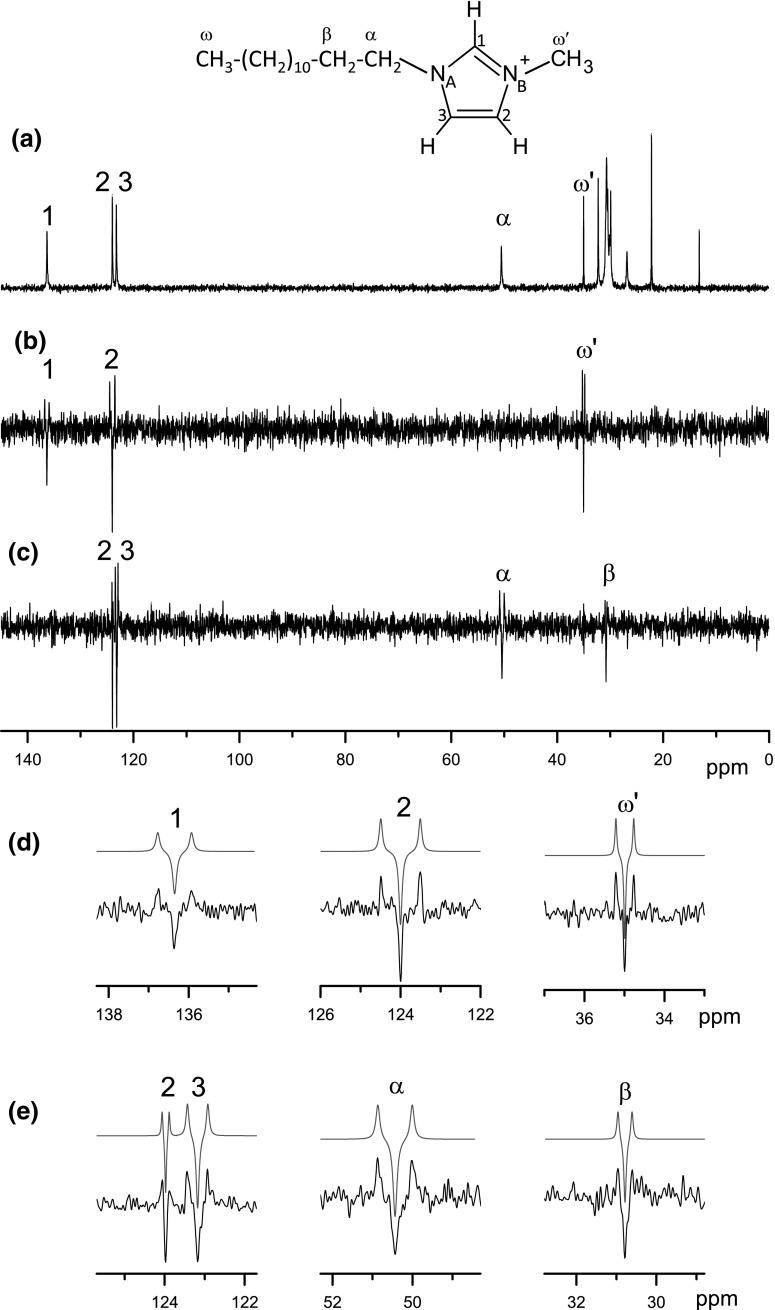
Carbon-13 spectra in the smectic A mesophase of the C12mimBF4 sample at 27 °C. **a** Single scan proton-decoupled carbon-13 CP spectrum. **b** ^13^C difference spectrum acquired with selective decoupling of nitrogen N_B_ in alternating scans. **c** ^13^C difference spectrum acquired with selective decoupling of nitrogen N_A_ in alternating scans. For difference spectra, 16k scans were accumulated with relaxation delay of 2s (9 h experimental time). In the parts **d** and **e**, multiplets from the spectra **b** and **c**, respectively, are displayed with expanded horizontal scale. Thin red lines show numerically simulated spectra with splitting values given in Table 1

In our study, however, heteronuclear couplings between two rare isotopes, carbon-13 and nitrogen-15, are measured. Carbon-13 spectra, recorded in alternated scans without and with decoupling of ^15^N spins, are subtracted. Strong dipolar interaction (in kHz range) with abundant ^1^H spins is suppressed by a high power proton decoupling applied in both spectra. Coupled carbon signals appear as doublets in the scans acquired without nitrogen decoupling, while, in the scans with ^15^N decoupling, they contribute to a residual central peak. The signals present in the difference spectrum are solely due to ^13^C–^15^N pairs, while the contribution of the carbons non-coupled to the nitrogen-15 spins is cancelled. The spectrum thus represents a superposition of the ^13^C–^15^N doublet and the central peak of opposite sign (Fig. 2). In this experiment, it is possible to observe even small splittings giving rise to satellites close to the central frequency, which, otherwise, would overlap with strong peaks due to ^13^C spins not coupled to ^15^N. We acquire ^13^C spectra rather than ^15^N spectra. The advantage is twofold. First, due to the higher Larmor frequency of carbon-13, the sensitivity of the registered signal is increased. Second, assignment of the couplings to spin pairs is simpler, since nitrogen atoms are fewer compared to carbons. Furthermore, sensitivity gain is obtained by cross-polarization (CP) of carbons from protons at Hartmann–Hahn condition. One disadvantage in using the difference approach is that every second scan contains no useful signal but contributes with noise. Hence, the noise level increases by a factor of 2^1/2^.

Since the resonance lines of two inequivalent nitrogen sites in imidazolium core are well separated in the nitrogen-15 spectrum (by about 11 ppm or 550 Hz, see Fig. 1), one can apply selective spin decoupling using a low-amplitude rf irradiation at the frequency of one nitrogen line without affecting the second line. The ^13^C difference spectrum with selective nitrogen decoupling contains only signals due to carbons coupled to irradiated nitrogen. In Fig. 2, the ^13^C difference spectra with selective nitrogen decoupling are shown and compared to the conventional ^13^C spectrum.

When the nitrogen N_B_ (right line in the NAN15 spectrum in Fig. 1) is selectively decoupled, only the signals of three directly bound carbons C1, C2, and *ω*′ are observed in the spectrum (the corresponding splittings obtained by numerical simulation of the spectral shapes, are given in Table 1). On the other hand, with decoupling of the nitrogen N_A_, the signals of C3 and *α* carbons are present, while signal of C1 carbon, which is also directly bound to N_A_, is missing in the spectrum. Interestingly, remote carbons C2 and *β* are also observed in this spectrum, giving rise to doublets with relatively small splitting.

**Table 1:** Splittings (in Hz) in the ^13^C NMR spectrum of C12mimBF4 due to couplings to nitrogen atoms in the imidazolium ring

	C1	C2	C3	*ω*′	*α*	*β*
N_A_	–	22	64	–	108	44
N_B_	106	124	–	56	–	–

The error bar is estimated to 5 Hz (0.1 ppm) based on numerical simulations of the experimental spectra (Fig. 2)

The splittings ∆*ν *= |2*D*_CN_ + *J*_CN_| are determined by the combined effect of the residual direct dipole–dipole heteronuclear interaction *D*_CN_ and indirect scalar coupling *J*_CN_. Values for one-bond *J*_CN_ coupling in the range from − 7 to − 17 Hz and for two-bond coupling in the range from − 1 to − 5 Hz have been reported for ionic derivatives of imidazole [30]. Hence, for the observed splittings, the dominant contribution is by dipolar interaction. Due to anisotropic molecular dynamics, orientation-dependent dipolar couplings in liquid crystals are scaled according to $D_{\text{CN}} = d_{\text{CN}} S_{\text{local}} (3\cos^{2} \theta_{NL} - 1)/2$, with $d_{\text{CN}} = - (\mu_{0} /8\pi^{2} )(\gamma_{\text{C}} \gamma_{\text{N}} \hbar /r_{12}^{3} )$ [22]. The local order parameter *S*_local_ describes the average orientation of internuclear vector with respect to liquid crystalline director *N*, and *θ*_*NL*_ is the angle between the director *N* and the laboratory frame axis *L* determined by the external magnetic field direction [22].

The absence of the C1 signal in the spectrum of Fig. 2c suggests that the splitting due to combined dipolar and *J*-coupling for the spin pair N_A_–C1 does not exceed the line width: |2*D*_CN_ + *J*_CN_| < 10 Hz. Assuming maximum *J*-coupling magnitude of 17 Hz [30], the dipolar coupling magnitude must be below 14 Hz. Small dipolar coupling can be explained, assuming that the average bond orientation with respect to the phase director is close to the magic angle leading to a low value of the local order parameter *S*_local_.

The resolution of the technique is limited by the carbon-13 line width, which, in the present study, is about 10 Hz. Additional ambiguity is due to the uncertainty of the relative signs of dipolar and *J*-couplings. Molecular motion and geometry considerations can be used to reveal the signs of the dipolar couplings and separate dipolar and *J*-coupling contributions. For this, details of complex anisotropic molecular rotations are required and they can be provided, for example, by MD simulation [31]. However, in the present *methodological* work, such an analysis was not attempted. In many thermotropic LC with higher molecular order, the *J*-coupling contribution is expected to be relatively small or negligible [20].

## Conclusion

Dipolar couplings are extensively exploited in studies of liquid crystals as a convenient and informative probe for different kinds of dynamic processes and structural properties. Commonly, couplings between abundant protons and rare isotopes, such as carbon-13, are measured using various SLF techniques [21, 22]. In this work, we have demonstrated an approach to record spectra of dipolar-coupled ^13^C–^15^N spin pairs in liquid crystalline samples with natural isotropic abundance. We show that, despite very low abundance of 0.004% for the naturally occurring ^13^C–^15^N pairs, sensitive and resolved spectra can be recorded within hours of experimental time using the conventional NMR instrument with moderately strong magnetic field of 11.7 T. Detection of carbon signal, instead of previously exploited nitrogen detection, increases the spectral sensitivity due to higher *γ*-ratio of ^13^C. Recording difference spectra with alternated nitrogen spin decoupling leads to the greater resolution of smaller splittings. While the technique can be extended to two-dimensional version to correlate multiple pair-wise spin couplings in a single 2D-experiment, relatively long experimental time makes 1D experiment more practical. Further gain in sensitivity can be achieved exploiting stronger magnets as well as NMR cryo-probes. The technique, which is demonstrated here for the thermotropic smectic phase of the ionic liquid, can be generally applied to other thermotropic or lyotropic mesophases.

## References

[1] Goossens K, Lava K, Bielawski CW, Binnemans K (2016). Chem. Rev..

[2] Fernandez AA, Kouwer PHJ (2016). Int. J. Mol. Sci..

[3] Axenov KV, Laschat S (2011). Materials.

[4] Kanazawa A, Ikeda T, Abe J (2001). J. Am. Chem. Soc..

[5] Tanabe K, Suzui Y, Hasegawa M, Kato T (2012). J. Am. Chem. Soc..

[6] Amann T, Dold C, Kailer A (2012). Soft Matter.

[7] Zhou Y, Antonietti M (2004). Chem. Mater..

[8] Dong RY (2010). Nuclear Magnetic Resonance Spectroscopy of Liquid Crystals.

[9] Fung BM (2002). Prog. Nucl. Magn. Reson. Spectrosc..

[10] Dvinskikh SV, Sandström D, Zimmermann H, Maliniak A (2006). Progr. Nucl. Magn. Reson. Spectrosc..

[11] Lesot P, Courtieu J (2009). Progr. Nucl. Magn. Reson. Spectrosc..

[12] Zimmermann H (1989). Liq. Cryst..

[13] Sandström D, Zimmermann H (2000). J. Phys. Chem. B.

[14] M.E. Neubert, in *Liquid Crystals: Experimental Study of Physical Properties and Phase Transitions*, ed. by S. Kumar (Cambridge University Press, Cambridge, 2001) Chapt. 10

[15] Witanowski M, Webb GA (1973). Nitrogen NMR.

[16] Cabane B, Gilbert Clark W (1970). Phys. Rev. Lett..

[17] Dvinskikh SV, Yamamoto K, Dürr UHN, Ramamoorthy A (2007). J. Magn. Reson..

[18] Kharkov BB, Chizhik VI, Dvinskikh SV (2012). J. Magn. Reson..

[19] Marini A, Zupancic B, Domenici V, Mennucci B, Zalar B, Veracini CA (2012). ChemPhysChem.

[20] Jackalin L, Dvinskikh SV (2017). Z. Phys. Chem..

[21] Hester RK, Ackerman JL, Neff BL, Waugh JS (1976). Phys. Rev. Lett..

[22] S.V. Dvinskikh, in *Modern Methods in Solid-State NMR: A practitioners’ Guide*, ed. by P. Hodgkinson (Royal Society of Chemistry, Abingdon, 2018)

[23] Cifelli M, Domenici V, Kharkov BB, Dvinskikh SV (2015). Mol. Cryst. Liq. Cryst..

[24] Fung BM, Khitrin AK, Ermolaev K (2000). J. Magn. Reson..

[25] Bertani P, Raya J, Bechinger B (2014). Solid State Nucl. Magn. Reson..

[26] Bak M, Rasmussen JT, Nielsen NC (2000). J. Magn. Reson..

[27] Lycka A, Dolecek R, Simunek P, Machacek V (2006). Magn. Reson. Chem..

[28] Fischbach I, Spiess HW, Saalwachter K, Goward GR (2004). J. Phys. Chem. B.

[29] Marchal JP, Canet D (1975). J. Am. Chem. Soc..

[30] Alei M, Morgan LO, Wageman WE, Whaley TW (1980). J. Am. Chem. Soc..

[31] Gerts ED, Komolkin AV, Burmistrov VA, Alexandriysky VV, Dvinskikh SV (2014). J. Chem. Phys..

